# Live, Genetically Attenuated, Cold-Chain-Free Cholera Vaccine—A Research and Development Journey: Light at the End of a Long Tunnel

**DOI:** 10.21315/mjms2022.29.2.1

**Published:** 2022-04-21

**Authors:** Manickam RAVICHANDRAN, Hui Xian TEW, Guruswamy PRABHAKARAN, Subramani PARASURAMAN, Mohd Nor NORAZMI

**Affiliations:** 1 Centre of Excellence for Vaccine Development (CoEVD), Faculty of Applied Sciences, AIMST University, Kedah, Malaysia; 2 Faculty of Applied Sciences, AIMST University, Kedah, Malaysia; 3 Centre of Excellence for Vaccine Development (CoEVD), Faculty of Pharmacy, AIMST University, Kedah, Malaysia; 4 School of Health Sciences, Universiti Sains Malaysia, Kelantan, Malaysia

**Keywords:** 5-aminolaevulinic acid (ALA) auxotroph, cholera, cold-chain-free, hemA mutation, live attenuated, vaccine

## Abstract

Cholera, a diarrheal disease caused by *Vibrio cholerae* (*V. cholerae*) O139 and O1 strains, remains a public health problem. The existing World Health Organization (WHO)-licenced, killed, multiple-dose oral cholera vaccines demand ‘cold-chain supply’ at 2 °C–8 °C. Therefore, a live, single-dose, cold-chain-free vaccine would relieve significant bottlenecks and costs of cholera vaccination campaigns. Our cholera vaccine development journey started in 2000 at Universiti Sains Malaysia with isolation of the *hem*A gene from *V. cholerae*, followed by development of a gene mutant vaccine candidate VCUSM2 against *V. cholerae* O139 in 2006. In 2010, VCUSM2 reactogenicity was reduced by replacing its two wild-type *ctx*A gene copies with mutated *ctx*A to produce strain VCUSM14. Introducing the *hem*A gene into VCUSM14 created VCUSM14P, a strain with the 5-aminolaevulinic acid (ALA) prototrophic trait and excellent colonisation and immunological properties (100% protection to wild-type challenged rabbits). It was further refined in Asian Institute of Medicine, Science and Technology (AIMST University), with completion of single- and repeated-dose toxicity evaluations in 2019 in Sprague Dawley (SD) rats, followed by development of a novel cold-chain-free VCUSM14P formulation in 2020. VCUSM14P is unique for its intact cholera toxin B, a known mucosal adjuvant. The built-in adjuvant makes VCUSM14P an ideal vaccine delivery platform for emerging diseases (e.g. severe acute respiratory syndrome coronavirus 2 [SARS-CoV-2] and tuberculosis). Our vaccine formulation mimics natural infection, remains non-reactogenic and immunogenic in vivo, and protects against infection and disease. It will also cost less and be less cumbersome to distribute due to its stability at room temperature. These features could revolutionise the outreach of this and other vaccines to meet global immunisation programmes, particularly in low-resourced areas. The next stage of our journey will be meeting the requisite regulatory requirements to produce the vaccine for rollout to countries where it is most needed.

## Introduction

Cholera is a diarrheal disease caused by the Gram-negative waterborne bacterium *Vibrio cholerae* (*V. cholerae*), an autochthonous inhabitant of aquatic environments, like rivers and seawater. The disease is transmitted by consuming contaminated food and drinking water. Left untreated, cholera can lead to dehydration and even death. It can be controlled by using clean drinking water, by adopting proper sanitation and by vaccination (1). According to the World Health Organization (WHO), nearly 80 countries across the globe reported cholera in 2020, with a total of 323,320 cases and 857 deaths, for a case-fatality rate of 0.27%. Yemen accounted for 85% of cholera cases reported worldwide (2) but the actual global number of cholera cases is uncertain because most cases go unreported (3). Cholera holds a stigma that has a direct negative impact on commercial trade and tourism, further discouraging reporting from many countries.

Among the 200 serogroups of *V. cholerae* recognised globally, the O1 and O139 serogroups are the ones known to cause cholera (1). The O139 serogroup, which first appeared in Chennai, India in October 1992, has spread to Bangladesh and other neighbouring countries (4). To date, 11 countries in Southeast Asia have reported the isolation of this serogroup. Although *V. cholerae* O1 is the predominant serogroup, the O139 serogroup has the potential to re-emerge as a significant cause of cholera (5). For these reasons, the focus of the present study is on developing a vaccine for cholera caused by the O139 serogroup of *V. cholerae*.

**Figure 1:**
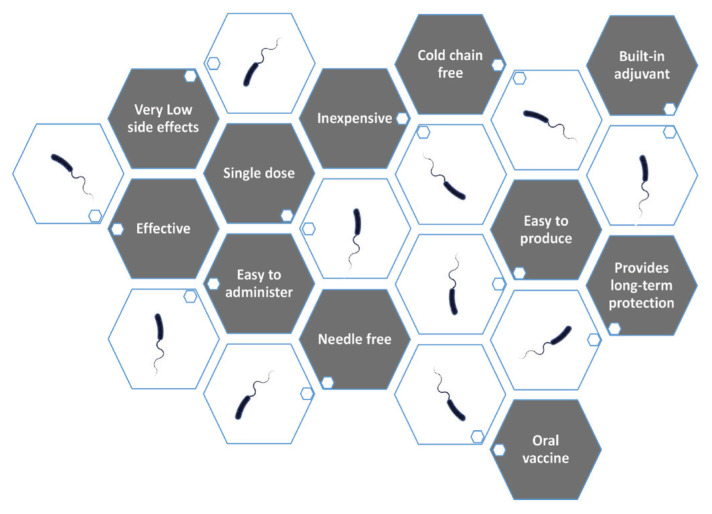
Our vaccine may have fulfilled all the criteria of an ideal vaccine

**Figure 2:**
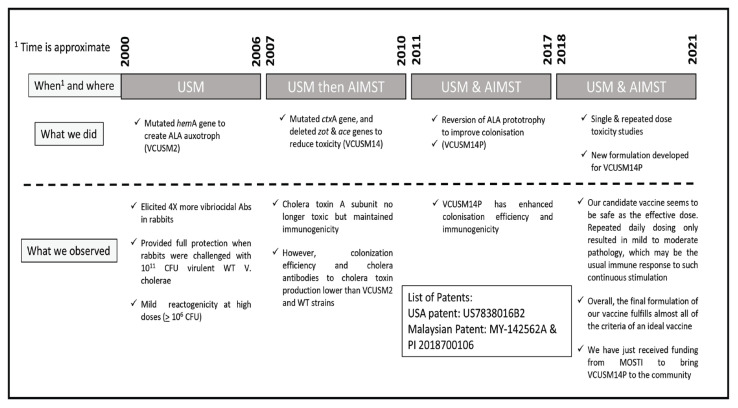
Our journey through time

Cholera is a disease of the poor; therefore, vaccine delivery and logistics must ensure that the people who most need the vaccine can receive it. The main targets of this study were to produce a vaccine with the following attributes: i) live attenuated (to ensure that only a single dose is needed); ii) good coloniser (to ensure it mimics the actual infection as closely as possible); iii) cold-chain-free (to reduce logistical problems of getting the vaccine to the recipients); iv) highly protective (a critical feature of any vaccines) and v) only mildly reactogenic (another critical feature of vaccines) (Figure 1). With the above targets in mind, our cholera vaccine development journey started. Details of this journey are described below and summarised in Figure 2.

## VCUSM2 Vaccine Strain Construction (Year 2000–2006)

We first mutated the *hem*A gene of *V. cholerae* (Genbank accession: AF227752) that encodes a key enzyme, glutamyl-tRNA reductase, used for the synthesis of 5-aminolaevulinic acid (ALA). This rendered the strain dependent on exogenous ALA for its growth. Experiments using adult rabbit models and infant mice showed that VCUSM2 was an excellent coloniser of the small intestine and elicited a greater than four-fold increase in vibriocidal antibodies in vaccinated rabbits. Rabbits immunised with VCUSM2 were fully protected against a subsequent challenge with 1 × 10^11^ colony forming units (CFU) of the virulent wild-type (WT) *V. cholerae* strain. Experiments using ligated rabbit ileal loops, which measure the level of fluid accumulation in the intestines following a challenge with VCUSM2, showed it to be 2.5-fold less toxic at the dose of 1 × 10^6^ CFU compared to the WT strain. Shedding of VCUSM2 in the rabbits (by studying the stool) occurred for no longer than four days, and the maximum survival rate of the attenuated vaccine in environmental water was eight days. By contrast, the WT strain survived for more than 20 days in environmental water. At this stage, we understood that our new strain caused mild reactogenicity in animal models at higher doses due to the cholera enterotoxin subunit A (*ctxA*) gene, which is the cholera toxin A subunit responsible for fluid secretion and diarrhoea, and due to the presence of the accessory cholera enterotoxin (*ace*) and zonula occludens toxin (*zot*) genes (6).

## VCUSM14 Vaccine Strain Construction (Year 2007–2010)

In the second phase of the study, we endeavoured to ensure that the reactogenicity was reduced to the very minimum. To do this, we modified the *ctx* operon using a systematic allelic replacement methodology to produce a mutant strain we designated VCUSM14. This strain has a mutated *ctx*A gene but an intact *ctx*B gene, whereas both the *zot* and *ace* genes were deleted. The mutation of the *ctx*A gene resulted in the retention of the immunogenicity of the cholera toxin A subunit (CTA) without producing toxicity. The amino acids arginine and glutamic acid at positions 7 and 112, respectively, in the CTA of VCUSM14 were substituted with lysine (R7K) and glutamine (E112Q). ELISA tests for cholera toxin using GM1 ganglioside, a receptor of CTA, showed that both WT CTA and mutated CTA were recognised by anti-CTA polyclonal antibodies. VCUSM14 produced comparatively less antigenic cholera toxin when compared to the parent VCUSM2 and the Bengal WT strain. However, VCUSM14 did not elicit fluid accumulation when inoculated into rabbit ileal loops at 10^6^ and 10^8^ CFU doses. The colonisation efficiency of VCUSM14 was one log lower than that of VCUSM2, which was attributed to the ALA auxotrophy and the less invasive properties of VCUSM14. VCUSM14 was therefore considered a non-reactogenic auxotrophic vaccine candidate against *V. cholerae* O139 (7).

## Construction of VCUSM14P: The Difficult Period (Year 2011–2017)

The challenge in this next phase was to increase the colonisation efficiency of VCUSM14. We had to go back one step to restore the intact *hem*A gene to revert the strain to an ALA prototroph. We used site-specific recombination to reincorporate the *hem*A gene into VCUSM14, and we named the resultant strain VCUSM14P. As expected, VCUSM14P had a colonisation ability almost equivalent to that of the WT O139 strain and showed a 68.5-fold increase in colonisation ability compared to VCUSM14, its parent strain. Tests with the reversible intestinal tie adult rabbit diarrhoea (RITARD) model confirmed that VCUSM14P showed no reactogenicity. In addition, histological analysis of the ileum showed no damage and the VCUSM14P bacteria were only detected on the endothelial surface of the mucosal layer. Importantly, a more than 300-fold increase was recorded in the anti-CT and anti-O139 LPS IgG titre and a greater than 300-fold and 1000-fold rise in vibriocidal antibody titre was recorded on the second and fourth week post-vaccination, respectively. The peak vibriocidal antibody titre was recorded on the third week post-vaccination, with a more than 2000-fold increase in antibody titre. The significant vibriocidal immune response indicated that VCUSM14P successfully induced an antibody response against the *V. cholerae* O139 serogroup. The immunised rabbits were protected when challenged with a lethal dose of the toxigenic O139 strain.

At this stage, we were happy that VCUSM14P showed great potential as a new non-toxic vaccine candidate against O139 *V. cholerae* capable of eliciting high antibody titres and protective immune responses. This phase of the study was a hidden period, however, as the manuscript describing these research findings was rejected by two prominent journals and we made no attempts to submit it to other potential journals. However, we proceeded to carry out the next phase of the research.

## Toxicity Evaluation (Year 2018–2020)

In the fourth phase of our research, we tested the single-dose and repeated dose toxicity of our VCUSM14P strain in Sprague Dawley (SD) rats to ensure its safety for eventual clinical use. The VCUSM14P strain did not show any single-dose toxicity signs at 1 × 10^7^ CFU. We then conducted a repeated dose toxicity study, involving administration of 30 repeated doses of cholera vaccine at three different concentrations (Group II: 1.25 × 10^6^ CFU; Group III: 2.5 × 10^6^ CFU and Group IV: 5 × 10^6^ CFU) to SD rats. The vaccine was administered orally to the SD rats at the desired dose every day, while normal saline was provided to the control group (Group I). No differences (*P* > 0.05) were observed in the SD rat body weights or biochemical parameters between the controls and the experimental SD rats after 15 and 30 repeated doses. However, significant increases (*P* < 0.05) in the organ to body weight ratios of the lungs, kidney, ureter, liver, heart and spleen were found in Groups II, III and IV compared to Group I. Haematological analysis revealed a significant increase in the white blood cell count in Groups II and IV compared to Group I. The histopathological findings suggested mild to moderate degeneration of the liver, kidney, heart and spleen of the treated SD rats. Mild lymphocyte infiltration in the lungs was observed in Groups II and III, and severe infiltration was observed in Group IV. These histopathological findings were possibly attributed to the 30 doses of vaccine given in daily succession without an interval. In the acute toxicity study, a single dose of the vaccine of up to 1 × 10^7^ CFU did not cause any harmful effects or lethality in SD rats (8). These findings indicated that the vaccine prototype was safe and did not cause any adverse effects or lethality in SD rats.

## Cold-Chain-Free Formulation (Year 2020)

In the final phase of our lab-based research, a prototype cold-chain-free live attenuated cholera vaccine formulation (LACV) was evaluated for its colonisation potential, immunogenicity, reactogenicity and protection after storage at room temperature for 140 days. In a suckling mouse colonisation assay, the formulated VCUSM14P showed 7.2 × 10^7^ CFU/mL compared to VCUSM14P (5.6 × 10^7^ CFU/mL) and the WT O139 strain (3.5 × 10^7^ CFU/mL). The formulation showed no reactogenicity in the rabbit ileal loop model, even at inoculation doses of 10^4^–10^6^ CFU/mL. Rabbits vaccinated with the LACV or unformulated VCUSM14P survived a challenge with wild-type O139 and showed no signs of diarrhoea or death in the RITARD model. Rabbits vaccinated with the formulated VCUSM14P showed a 275-fold increase in anti-CT IgG and a 15-fold increase in anti-CT IgA antibodies compared to rabbits vaccinated with the unformulated VCUSM14P. Vibriocidal antibodies were increased by 31-fold with the LACV and 14-fold with unformulated VCUSM14P. The vaccine formulation mimicked a natural infection, was non-reactogenic and highly immunogenic in vivo and protected animals from a lethal WT *V. cholerae* O139 challenge. The single-dose LACV formulation was stable at room temperature (25 ± 2 °C) for 140 days, indicating that it would result in significant cost savings during mass cholera vaccination campaigns (9).

We were therefore on our way to developing an ideal vaccine (Figure 1) against cholera. The vaccine also has the potential for use as a delivery vehicle for other vaccines, especially against pathogens that enter via the mucosal route.

## Highlights of the Vaccine Prototype

One of the unique features of VCUSM14P is that it has an intact cholera toxin (CTB), which is a known mucosal adjuvant. VCUSM14P is a non-toxigenic strain and has a built-in adjuvant (CTB); therefore, it can be used as an efficient vaccine delivery platform for diseases that enter the mucosa, such as severe acute respiratory syndrome coronavirus 2 (SARS-CoV-2) and tuberculosis. Developing an affordable cold-chain-free oral vaccine could provide excellent stimulation of the mucosal immune response. In brief, our vaccine formulation mimics a natural infection and is highly immunogenic and non-reactogenic. It protects animals from a lethal WT *V. cholerae* O139 challenge and costs less due to its stability at room temperature, making it ideal for global immunisation programmes.

## Challenges and the Way Forward

**Figure 3:**
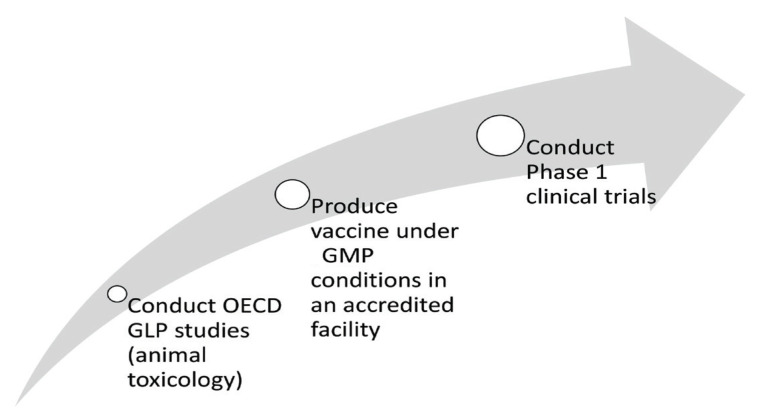
Next stages of our journey to be funded by the Ministry of Science, Technology and Innovation (MOSTI)

Our journey has now entered the challenging stages of bringing our vaccine to clinical development (Figure 3). Prior to undergoing the three clinical stages of development, the vaccine needs to be tested in a ‘Good Laboratory Practice’ (GLP) animal toxicity study under strict international regulatory guidelines, followed by the production of the vaccine in a ‘Good Manufacturing Practice’ (GMP) facility in compliance with international guidelines. At present, GMP production and all three clinical phases of development have to be performed outside Malaysia. We do not currently have a GMP facility for bacterial fermentation for human use. Furthermore, we are still not ready to run the trials for biologics for a Phase I clinical trial, and we cannot run Phase 2 and 3 trials in Malaysia since the number of cholera cases is smaller than in other endemic countries. Thus, from this point on, bringing our vaccine to further developmental stages will be expensive and will require strong support from the government and industries. This vaccine platform is a live bacterial type, cold-chain-free and relatively inexpensive to manufacture; therefore, it will be the vaccine prototype for the bottom billions of the world. This may make it less appealing for investors, since the return on investment may not be sufficiently attractive for them. Nonetheless, our motivation is to ensure that our vaccine will be helpful to those who need it most.

Our long journey is now entering the exciting phases of development to finally (we hope) reach the community. A couple of lessons learnt during this long and difficult journey are worth mentioning. One is that, despite several grants that we successfully acquired, several more of our proposals have been rejected. We faced a variety of review panel members, some of whom are not working in this area of expertise (technically or procedurally) or are not visionary enough, and this may have stalled our journey, making it longer than desired. The tracking of research success is purely a numbers game, rather than having a strong basis in science and innovation. The second difficulty that we faced was the lack of good students to take up the challenge of the project. Furthermore, the way research is run in a university setting has perhaps inadvertently made the journey longer than necessary. It was (and still is) a ‘two steps forward, one step back’ journey, as every time a postgraduate student graduates, we have to train a new student, sometimes from the very beginning, before the project can move forward again. Keeping the same core team members together has also been a challenge. We hope our resilience will finally pay off, as we assemble a core team for the way forward.

Our vaccine prototype is currently being tested for its efficacy against enterotoxigenic *E. coli* (ETEC) diarrhoea in an animal model and as a DNA vaccine delivery vector for immunisation against COVID-19 and tuberculosis.

## References

[1] Almagro-Moreno S, Taylor RK (2013). Cholera: environmental reservoirs and impact on disease transmission. Microbiol Spectr.

[2] World Health Organization (WHO) (2021). Cholera, 2020. Weekly Epidemiological Record.

[3] Ali M, Nelson AR, Lopez AL, Sack DA (2015). Updated global burden of cholera in endemic countries. PLoS Negl Trop Dis.

[4] Albert MJ (1996). Epidemiology and molecular biology of *Vibrio cholerae* O139 Bengal. Indian J Med Res.

[5] Chowdhury F, Mather AE, Begum YA, Asaduzzaman M, Baby N, Sharmin S (2015). *Vibrio cholerae* serogroup O139: isolation from cholera patients and asymptomatic household family members in Bangladesh between 2013 and 2014. PLoS Negl Trop Dis.

[6] Ravichandran M, Ali SA, Rashid NHA, Kurunathan S, Yean CY, Ting LC (2006). Construction and evaluation of a O139 *Vibrio cholerae* vaccine candidate based on a hemA gene mutation. Vaccine.

[7] Chan M, Cheong TG, Kurunathan S, Chandrika M, Ledon T, Fando R (2010). Construction and characterisation of an auxotrophic ctxA mutant of O139 *Vibrio cholerae*. Microb Pathog.

[8] Xian TH, Parasuraman S, Sinniah K, Ravichandran M, Prabhakaran G (2019). Repeated dose toxicity evaluation of a cold chain-free, live, attenuated oral cholera vaccine in Sprague Dawley rats. Vaccine.

[9] Xian TH, Sinniah K, Yean CY, Krishnamoorthy V, Bahari MB, Ravichandran M (2020). Immunogenicity and protective efficacy of a live, oral cholera vaccine formulation stored outside-the-cold-chain for 140 days. BMC Immunol.

